# A Cross-Sectional Study on 3-(2-Deoxy-β-D-Erythro-Pentafuranosyl)Pyrimido[1,2-α]Purin-10(3H)-One Deoxyguanosine Adducts among Woodworkers in Tuscany, Italy

**DOI:** 10.3390/ijms20112763

**Published:** 2019-06-05

**Authors:** Filippo Cellai, Fabio Capacci, Carla Sgarrella, Carla Poli, Luciano Arena, Lorenzo Tofani, Roger W. Giese, Marco Peluso

**Affiliations:** 1 Cancer Factor Risk Branch, Regional Cancer Prevention Laboratory, Institute for Cancer Research, Prevention and Clinical Network (ISPRO), 50139 Florence, Italy; f.cellai@ispro.toscana.it; 2 Functional Unit for Prevention, Health and Safety in the Workplace, ASL10, 50139 Florence, Italy; fabio.capacci@uslcentro.toscana.it (F.C.); carla.sgarrella@uslcentro.toscana.it (C.S.); 3 Department of Prevention, Azienda USL Toscana Centro, 50139 Florence, Italy; carla.poli@uslcentro.toscana.it (C.P.); luciano.arena@uslcentro.toscana.it (L.A.); 4 Department of Neurosciences, Psychology, Drug Research and Child Health, University of Florence, 50139 Florence, Italy; lorenzo120787@gmail.com; 5 Bouve College of Health Sciences, Barnett Institute, Northeastern University, Northeastern University, Boston, MA 02115, USA; r.giese@northeastern.edu

**Keywords:** wood dust, nasal epithelia, VOCs, M_1_dG

## Abstract

Occupational exposure to wood dust has been estimated to affect 3.6 million workers within the European Union (EU). The most serious health effect caused by wood dust is the nasal and sinonasal cancer (SNC), which has been observed predominantly among woodworkers. Free radicals produced by inflammatory reactions as a consequence of wood dust could play a major role in SNC development. Therefore, we investigated the association between wood dust and oxidative DNA damage in the cells of nasal epithelia, the target site of SNC. We have analyzed oxidative DNA damage by determining the levels of 3-(2-deoxy-β-D-erythro-pentafuranosyl)pyrimido[1,2-α]purin-10(3H)-one deoxyguanosine (M_1_dG), a major-peroxidation-derived DNA adduct and a biomarker of cancer risk in 136 woodworkers compared to 87 controls in Tuscany, Italy. We then examined the association of M_1_dG with co-exposure to volatile organic compounds (VOCs), exposure length, and urinary 15-F_2t_ isoprostane (15-F_2t_-IsoP), a biomarker of oxidant status. Wood dust at the workplace was estimated by the Information System for Recording Occupational Exposures to Carcinogens. M_1_dG was measured using ^32^P-postlabeling and mass spectrometry. 15-F_2t_-IsoP was analyzed using ELISA. Results show a significant excess of M_1_dG in the woodworkers exposed to average levels of 1.48 mg/m^3^ relative to the controls. The overall mean ratio (MR) between the woodworkers and the controls was 1.28 (95% C.I. 1.03–1.58). After stratification for smoking habits and occupational status (exposure to wood dust alone and co-exposure to VOCs), the association of M_1_dG with wood dust (alone) was even greater in non-smokers workers, MR of 1.43 (95% C.I. 1.09–1.87). Conversely, not consistent results were found in ex-smokers and current smokers. M_1_dG was significantly associated with co-exposure to VOCs, MR of 1.95 (95% C.I. 1.46–2.61), and occupational history, MR of 2.47 (95% C.I. 1.67–3.62). Next, the frequency of M_1_dG was significantly correlated to the urinary excretion of 15-F_2t_-IsoP, regression coefficient (β) = 0.442 ± 0.172 (SE). Consistent with the hypothesis of a genotoxic mechanism, we observed an enhanced frequency of M_1_dG adducts in woodworkers, even at the external levels below the regulatory limit. Our data implement the understanding of SNC and could be useful for the management of the adverse effects caused by this carcinogen.

## 1. Introduction

Occupational exposure to wood dust has been estimated to affect 3.6 million workers within the European Union (EU) [1]. Among these, 1.5 million workers are exposed to low dust levels (<0.5 mg/m^3^), whereas 0.2 million workers are exposed to higher dust values (>5 mg/m^3^). The most serious health effect caused by wood dust is nasal and sinonasal cancer (SNC) [2], which has been observed predominantly among woodworkers. In 1960, the first link between SNC and wood dust was found in the British furniture industry [3]. In that study, a several hundred-fold higher risk of SNC was detected among woodworkers relative to unexposed controls. In 1995, based on epidemiological evidence, the International Agency for Research on Cancer (IARC) classified wood dust as a human carcinogen (Group 1) (IARC, 1995). SNC is a rare neoplasm, accounting for 3% of head and neck cancers, with squamous cell carcinoma and adenocarcinoma (AD) histotypes, where AD is more frequently correlated to wood dust. In a recent meta-analysis [4], the relative risk for SNC among woodworkers was estimated to be of 5.91 (95% Confidence Interval (C.I.) 4.31–8.11) in case-control studies and 1.61 (95% C.I. 1.10–2.37) in cohort studies. In that meta-analysis, the highest risk was observed for the AD subtype (29.43, 95% C.I. 16.46–52.61). The overall incidence of SNC was estimated at 0.5 cases per 100,000 between 1978–2002 in the EU [4]. Whereas the incidence of SNC was at 0.4–2.0 per 100,000 in men and at 0.1–0.5 per 100,000 in women in the period 1998–2002 in Italy. A significant risk of SNC was found in the wood and furniture industry in the Piedmont region, Italy, Odds Ratio (OR) of 4.4 (95% C.I. 1.41–13.4), and in Tuscany, Italy, OR of 5.4 (95% C.I. 1.7–17.2) [4]. The latter investigation showed the greatest correlation between wood dust and the AD subtype, OR of 89.7 (95% C.I. 19.8–407.3). In another case-control study in Piedmont, Italy [4], a significant risk of SNC was found to be associated with ever exposure to wood dust, OR of 11.4 with a risk for AD, OR of 58.6, ten-fold greater than for other histotypes. Moreover, the risk for AD subtype doubled every five-years of occupational history.

Free radicals released during inflammatory response to wood dust could play a major role in SNC development [5]. However, the genotoxic effects caused by inflammatory reactions subsequent to wood dust exposure are poorly known. Excessive radical oxygen species (ROS) induced by inflammation [6] can damage protein, membrane lipids, and genetic DNA [7]. In particular, the oxidative degradation of lipids by a free radical chain reaction mechanism can result in their non-enzymatic degradation to many compounds, including 4-hydroxynonenal and malondialdehyde, the latter a reactive aldehyde [8], capable of interacting with DNA to give 3-(2-deoxy-β-D-erythro-pentafuranosyl)pyrimido[1,2-α]purin-10(3H)-one deoxyguanosine (M_1_dG) adducts [9]. M_1_dG adducts, if not completely repaired, can block cellular replication and cause base pair and frameshift mutations [10]. M_1_dG adducts have been associated to DNA methylation aberrations in the Long Interspersed Nuclear Element-1 repeated sequences and in the inflammatory cytokine interleukin(IL)-6 gene [11,12]. DNA aberrations and mutations are important signs of carcinogenic process and hospital-based studies showed that high M_1_dG levels are linked to cancer development and tumor progression [13,14,15].

Significant associations between occupational air pollution exposure and increments of biomarker exposure and cancer risk are apparent [16,17,18,19]. Recently, we have examined the prevalence of urinary 15-F_2t_ isoprostane (15-F_2t_-IsoP), a biomarker of oxidant status [20], in woodworkers compared to controls in Tuscany, Italy [21]. In that study, we found a significant excess of urinary 15-F_2t_-IsoP excretion in woodworkers with an overall Means Ratio (MR) of 1.36, 95% C.I. 1.18–1.57. Subsequently, we wondered whether woodworkers experienced enhanced frequency of M_1_dG adducts, a biomarker of oxidative stress and cancer risk [15,18,19,22,23,24], which could indicate potential health risks in later life. Therefore, we have examined the frequency of M_1_dG in the cells of the nasal epithelia, the target site of SNC [5]. Our approach consisted in the conduction of a cross-sectional study to evaluate the prevalence of M_1_dG, derived from peroxidation of DNA, in 136 woodworkers compared to 87 unexposed controls in Italy’s Tuscany region. We then examined the association of M_1_dG with co-exposure to volatile organic compounds (VOCs), exposure length, and the urinary levels of 15-F_2t_-IsoP. Wood dust exposure was estimated by the Information System for Recording Occupational Exposures to Carcinogens (SIREP) [25]. SIREP is a system that provides the data that have been measured by employers in industry atmosphere according to Italian laws. M_1_dG was examined by ^32^P-postlabeling [26] and mass spectrometry [27]. Urinary 15-F_2t_-IsoP data were obtained from a previous study [21]. Additional understanding of the link between M_1_dG adducts and wood dust exposure can improve the knowledge of the mechanisms of actions of this carcinogen in the cells of the respiratory tract of woodworkers.

## 2. Results

### 2.1. Demographic Variables

Out of the 44 local wood companies contacted by medical doctors and local health services, 32 consented to participate in this study. There were 136 woodworkers—50 smokers and 25 ex-smokers (mean age 45.2 years ± 11 Standard Deviation (SD))—and 87 unexposed controls—26 smokers and 11 ex-smokers (mean age 45.6 years ± 9 SD). All participants were males, because most woodworkers are men. Controls were resident in areas of the Tuscany region with no proximity to major air pollutant sources. Demographic characteristics and lifestyle habits of workers and controls were comparable.

### 2.2. Wood Dust Exposure

The airborne levels of wood dust were quantified by an 8-h time-weighted average (TWA-8), according to Italian laws [25]. Wood dust values corresponded to a single value assessed from several consecutive samples obtained by fixed positions located at each workplace. The mean daily concentrations of airborne wood dust were of 1.48 mg/m^3^ in the wood industries that participated in this study.

### 2.3. Reference M_1_dG Adduct Standards by ^32^P-Postlabeling and/or Mass Spectrometry

The levels of M_1_dG, expressed as RAL, were 5.0 M_1_dG adducts ± 0.6 (Standard Error (SE)) per 10^6^ nn in the MDA-treated calf-thymus (DNA by ^32^P-DNA post labeling) [26]. The presence of M_1_dG in the MDA-treated calf-thymus DNA sample was confirmed by the matrix-assisted laser desorption/ionization time-of-flight mass spectrometry [27]. Relative to the accurate masses that were found in the spectrum from one spot, the exact mass of M_1_dG was as follows, using the M nomenclature: M_1_dG (581.166). A calibration curve was set up by diluting this reference DNA adduct standard sample with untreated calf-thymus DNA and measuring the decreasing levels of M_1_dG, r-squared = 0.99.

### 2.4. M_1_dG and Wood Dust

To investigate of the potential genotoxic of wood dust, we analyzed the levels of M_1_dG using ^32^P-postlabeling [26]. A characteristic profile of M_1_dG adduct spot was detected in the chromatographic plates of the study population. The visual intensity of the M_1_dG adduct spot was generally stronger in the plates of the woodworkers compared to the controls. When we analyzed the frequency of M_1_dG in the woodworkers, our findings showed that the adduct frequency was significantly higher, up to 1.7-fold, within the workers exposed to wood dust as compared to the controls. Table 1 reports that there were 83.2 and 50.4 M_1_dG adducts per 10^8^ nn among the woodworkers and the controls, respectively. The overall mean ratio (MR) between the woodworkers and the unexposed controls was 1.28 (95% C.I. 1.03–1.58). Current smokers had also average amounts of adducts greater than ex-smokers and non-smokers, but the difference was not statistically significant. A significant relationship between adducts and occupational history was detected (147% excess). After stratification for both smoking habits and exposure status (exposure to wood dust alone and co-exposure to VOCs), results in Table 2 show that the relationship of M_1_dG with wood dust (alone) was even greater in non-smokers workers, MR of 1.43 (95% C.I. 1.09–1.87). However, inconsistent results were found in ex-smokers and current smokers. Instead, M_1_dG was significantly associated with a co-exposure to VOCs, MR of 1.95 (95% C.I. 1.46–2.61), and occupational history, MR of 2.47 (95% C.I. 1.67–3.62). The highest amount of M_1_dG was measured in woodworkers who reported to be smokers as well as co-exposed to airborne organic solvents (158.4 M_1_dG adducts per 10^8^ nn) during woodworking.

### 2.5. M_1_dG and 15-F_2t_ Isoprostane

Next, we examined the correlation between the levels of M_1_dG adducts with the urinary concentrations of 15-F_2t_-IsoP, a biomarker of oxidant status [20], among woodworkers. Multivariate regression analysis showed that the M_1_dG adducts were linearly correlated to the urinary excretion of 15-F_2t_-IsoP, regression coefficient (β) = 0.442 ± 0.172 (SE), *p*-value = 0.011.

## 3. Discussion

In the current study, we examined the genotoxic effects associated with wood dust exposure using a comparative cross-sectional study, with larger numbers of subjects than prior studies [28,29,30]. The indoor concentrations of wood dust were used as a marker of external carcinogen exposure in the 32 wood companies located in Italy’s Tuscany Region that participated in the study. The European Union Directive (1999/38) has classified wood dust as a carcinogenic agent and has set the occupational exposure limit (OEL) to 5.0 mg/m^3^. The Scientific Committee for Occupational Exposure Limits (SCOEL) of the European Union has stated that exposure to wood dust above 0.5 mg/m^3^ can cause pulmonary effects and should be avoided at workplace [1]. The air monitoring results, which were obtained from the SIREP system, showed that the indoor average levels of wood dust at the workplace was 1.48 mg/m^3^.

Multivariate regression analysis showed that the frequency of M_1_dG in exposed woodworkers was significantly enhanced as compared to the unexposed controls, MR of 1.28 (95% C.I. 1.03–1.58), even though the airborne exposure to wood dust was below the Italian regulatory limit of 5.0 mg/m^3^ (Legislative Decree No 66/2000). The correlation of M_1_dG with dust becomes even stronger in non-smokers who were exposed to wood dust (alone), MR of 1.43 (95% C.I. 1.43–1.87), after stratification for smoking habits and co-exposure status. Inconsistent results were found in ex-smokers and current smokers where the genotoxic effects of wood dust (alone) could be potentially merged with tobacco smoke carcinogens. Instead, strong associations were observed with co-exposure to VOCs, that reached the statistical significance among non-smokers and current smokers, MR of 1.59 (95% C.I. 1.05–2.43) and 2.33 (95% C.I. 1.45–3.70), respectively. In doing so, M_1_dG generation was found to be significantly correlated to occupational history, MR of 2.47 (95% C.I. 1.67–3.62) and 15-F_2t_-IsoP urinary excretion (*p*-value of 0.011).

The significant increment of M_1_dG in woodworkers can be caused by ROS released during inflammatory reactions as a consequence of exposure to fine and abundant airborne dust created during woodworking. Significant evidence for inflammations as a consequence of dust exposure comes from an earlier study with experimental animals [31]. In that experiment, repeated airway exposure to wood dust induced inflammation, which was accompanied by several proinflammatory cytokines and chemokines, in the lungs of mice. During the inflammatory response, activated macrophages and neutrophils generate a variety of highly reactive oxidants, such as hydrogen peroxide and hypochlorite acid [32], which are capable of reacting with lipids of membrane leading to reactive aldehydes, which are further capable of interacting with DNA forming M_1_dG adducts [7]. Our findings are in line with previous studies that report increased biomarkers in various tissues, such as peripheral blood and oral cells, of workers exposed to wood dust by micronucleus and comet (single-cell gel electrophoresis) techniques [28,29,30]. In those studies, high DNA strand breaks [28,29,30] and enhanced chromosomal instability values [2,28,33] were found in the woodworkers relative to the unexposed controls, but discrepant findings were also shown [34]. Results also provide evidence of a significant M_1_dG increment (147% excess) in the long-term workers compared to those with shorter exposure, used as a reference level. Long-term adverse genotoxic effects due to inflammations and ROS production cannot be excluded in cells of respiratory epithelia. These findings are in line with previous studies [28,35]. For instance, the generation of a urinary biomarker of oxidative stress was found to be significantly correlated with the length of exposure in a study of workers occupationally exposed to asbestos. Rekhadevi et al. [28] found a significant association between the frequency of micronuclei and the length of occupational exposure to wood dust. Taken together, our results indicate that the production of aldehydes by oxidative degradation of lipids of cellular membranes can represent an obvious possible mechanism that could explain wood carcinogenicity.

Next, we observed a consistent increment of M_1_dG (95% excess) with exposure to VOCs, another established risk factor in woodworking [36]. VOCs, such as benzene, xylene, and formaldehyde, are commonly used in wood companies, in particular by subjects involved in varnishing and cleaning of furniture elements [36]. This result is not surprising because exposure to benzene, toluene, and xylene is correlated to the production of ROS, heat shock proteins, and oxidative stress [37]. Benzene is a chemical that can be metabolized by CYP_2_E*_1_* to various chemicals with the capability of redox-cycling, a reaction that generates ROS [38]. Formaldehyde is a substrate of CYP_2_E_2_ and can be oxidized by peroxidase, aldehyde oxidase, and xanthine oxidase with ROS formation [39]. Formaldehyde exposure has been associated with high levels of CYP_1_A_1_ and GSH and GSTT_1_ downregulation [40].

15-F_2t_-IsoP is a widely used biomarker of exposure [21,41]. Therefore, it is of primary importance to understand its biological relevance in terms of health risk. Recently, an early study has supported the hypothesis of a relationship between F_2_-IsoP and 8-hydroxy-2′-deoxyguanosine, a sensitive marker of oxidative stress and cancer risk [24], in experimental animals [42]. Therefore, we have evaluated the association between 15-F_2t_-IsoP and M_1_dG in our study population. Results show that the levels of M_1_dG were statistically significantly correlated with the urinary excretion of 15-F_2t_-IsoPs, indicating that 15-F_2t_-IsoP is significantly linked to the induction of early cancerogenic effects in the cells of nasal epithelia, the target site of SNC [5].

Although exposure registries are commonly used for the purposes of hazard control, exposure surveillance, and assessment of health risks [1], such the SIREP system [25], this approach has few limitations, including potential exposure misclassification rising from the heterogeneity of wood dust exposure levels within different wood industries. This does not completely reflect the exposure status of each woodworker. Indeed, there could be an underestimation of carcinogen exposure associated with some working operations [43]. Unreported variations in the use of the Personal Protective Equipment [44] could have influenced the individual levels of exposure to wood dust. In addition, a weakness of the study is that exposure levels to wood dust were measured with stationary stations and not personal samplers. Measurements from fixed samplers provide evidence of woodworkers’ exposure via air but they are not well representative of individual exposures to dust due to spatial and temporal variations. Among the strengths of this study was the use of exfoliated nasal epithelia cells for adduct analysis, which consists of 89% of epithelial cells and 11% of neutrophils, with few eosinophils and lymphocytes. Unfortunately, we did not measure cell abnormalities in nasal epithelial cells in woodworkers to examine potential correlation between M_1_dG levels and pre-cancerous lesions.

## 4. Material and Methods

### 4.1. Study Population

In this cross-sectional study, we randomly selected 44 wood industries among those which were under mandatory surveillance for assessing workplace health risks in Italy’s Tuscany region. Companies were contacted by occupational physicians working in the local health service. Eligibility criteria were as follows: (1) to be employed in companies of the Florence province from at least one year (for the workers) and (2) to not have history of occupational or environmental carcinogen exposures (for the controls). All the participants were informed about the aims of the study and provided a written informed consent. Details about age, gender, professions, residence, lifestyle habits, occupational status and history, including exposures to carcinogens, such as VOCs and formaldehyde, were obtained by questionnaire. The study was approved by the Institutional Review Board of the General Hospital.

### 4.2. Wood Dust Exposure Measurement

Data contained in SIREP system [25] were used to compute the indoor concentrations of wood dust in the local wood companies. These data on carcinogen exposure were measured by fixed station air samplers at the workplace, collected by employers and regularly sent to the exposure registers, which contains quantitative measurements of volatile wood dust exposure.

### 4.3. Nasal Epithelia Brushing

Brushing is an easy and relatively noninvasive method to collect exfoliated cells from nasal epithelia [13]. Briefly, cells of the respiratory tract were collected from clean lower turbinate in each nostril with a cytobrush in the morning at workplace. Brushing samples were then treated with 10% acetylcysteine to break down mucus gel structure. After centrifugations, cellular pellets were stored at −80 °C.

### 4.4. Reference Adduct Standard

Calf-thymus DNA was exposed to 10 mM MDA (ICN Biomedicals, Irvine, CA, USA), as described [22]. MDA-treated calf-thymus DNA was diluted with untreated DNA to obtain lower amounts of the reference adduct standard to generate a calibration curve.

### 4.5. DNA Extraction and Purification

DNA was extracted and purified by digestion with ribonucleases A and T_1_, proteinase K treatment, extraction with organic solvents and ethanol precipitation [45]. DNA concentration and purity of biological samples were determined by spectrophotometry. Coded DNA samples were stored at −80 °C until laboratory analysis.

### 4.6. Mass Spectrometry

The generation of M_1_dG in MDA-treated calf-thymus DNA was analyzed by mass spectrometry (Voyager DE STR from Applied Biosystems, Framingham, MA), as previously reported [27].

### 4.7. ^32^P-DNA Postlabeling

M_1_dG formation was examined by ^32^P-postlabeling [26]. In detail, DNA (2 μg) was incubated with micrococcal nuclease (21.4 mU/μL) and spleen phosphodiesterase (6.0 mU/μL) in hydrolysis buffer, pH 6.0 at 37 °C for 4.5 h. Digests were treated with nuclease P1 (0.1 U/μL) at 37 °C for 30′ [46]. Nuclease P1-resistant nucleotides were incubated with 25 μCi of carrier-free [γ-^32^P]-ATP (3000 Ci/mM) and polynucleotide kinase T_4_ (0.75 U/μL) to generate ^32^P-labeled adducts in bicine buffer, pH 9.0, at 37 °C for 30 min. The generation of M_1_dG was analyzed using the following chromatography system: MgCl_2_ (0.35 M) for preparatory chromatography; while 2.1 M lithium formate, 3.75 M urea pH 3.75 and 0.24 M sodium phosphate, 2.4 M urea pH 6.4, were used for the bidimensional chromatography, respectively. M_1_dG was detected by storage phosphor imaging employing intensifying screens, which were scanned with Typhoon 9210 (Amersham). The relative adduct labelling (RAL) of M_1_dG was calculated by the following formula = (pixels in adducted nucleotides)/(pixels in nn). Adduct values were computed considering the recovery of the reference adduct standard.

### 4.8. Statistical Analysis

M_1_dG levels were reported per 10^8^ nn. RAL values were log transformed before performing the statistical analyses. Log-normal regression models, with age (years), smoking habits (never, ex, and current), and occupational status (woodworker vs. unexposed control) and history (years), such as independent variables, were employed to analyze the correlation of wood dust exposure with M_1_dG. Workers were then sub-grouped according to VOC co-exposures in: (1) exposed to wood dust alone and (2) co-exposed to VOCs. MR estimates and its 95% C.I. were used as a measure of effect [47] for each level of the predictor variables relative to the control group. Statistical analysis was done using the software SAS9.3 and SPSS 20.0 (IBM SPSS Statistics, New York, NY, USA).

## 5. Conclusions

Our study adds relevant data to the body of literature, originating from population-based studies, showing significant induction of cellular anomalies indicative of genotoxicity in the target site of SNC, even at airborne levels of wood dust below the Italian regulatory limit. Consistent with a genotoxic mechanism, we observed an enhanced frequency of M_1_dG adducts in the cells of the respiratory tract of workers occupationally exposed to wood dust. Certainly, other aldehyde component could contribute to the total adduct formation, which could result in a more complex pattern of genotoxicity. Worker surveillance using biomarkers of exposure and cancer risk, such as end points, could be relevant for managing health and safety risk of woodworkers.

## Figures and Tables

**Table 1:** Mean level of three-(2-deoxy-β-D-erythro-pentafuranosyl)pyrimido[1,2-α]purin-10(3H)-one deoxyguanosine (M_1_dG) adducts, expressed as a relative adduct level (RAL) per 10^8^ normal nucleotides (nn), Mean Ratio (MR), and 95% Confidence Interval (C.I.), by exposure to wood dust and other variables.

M_1_dG, Smoking Habits, Exposure to Wood Dust, and Occupational History				
	N	RAL Per 10^8^ nn ± Standard Error	Mean Ratio and 95% C.I.	*p*-Value ^a^
Smoking habits				
Non-smokers	111	63.7 ± 4.7	1	
Former smokers	36	68.7 ± 9.6	0.97 (95% C.I. 0.73–1.31)	0.873
Current smokers	76	81.0 ± 8.5	1.17 (95% C.I. 0.92–1.48)	0.190
Exposure to wood dust				
Controls	87	50.4 ± 2.7	1	
Woodworkers	136	83.2 ± 6.2	1.28 (95% C.I. 1.03–1.58)	0.021
Occupational history				
≤ 8 years	40	67.9 ± 15.1	1	
9–25 years	49	79.0 ± 8.8	1.67 (95% C.I. 1.16–2.38)	0.005
≥ 26 years	47	100.6 ± 8.2	2.47 (95% C.I. 1.67–3.62)	<0.001

^a^*p*-values (Test of Wald) were adjusted for age and smoking, as appropriate.

**Table 2:** Average level of three-(2-deoxy-β-D-erythro-pentafuranosyl)pyrimido[1,2-α]purin-10(3H)-one deoxyguanosine (M_1_dG), expressed as relative adduct level (RAL) per 10^8^ normal nucleotides, Mean Ratio (MR), and 95% Confidence Interval (C.I.), by smoking habits and occupational status, i.e., exposure to wood dust alone and co-exposure to volatile organic compounds (VOCs).

M_1_dG, Exposure to Wood Dust (Alone), and Co-Exposure to VOCs				
	N	RAL per 10^8^ nn ± Standard Error	Mean Ratio and 95% C.I.	*p*-Value ^a^
Non-smokers				
Controls	50	45.5 ± 3.4	1	
Workers exposed to wood dust (alone)	48	78.8 ± 8.6	1.43 (95% C.I. 1.09–1.87)	0.009
Woodworkers exposed to VOCS	13	80.3 ± 17.5	1.59 (95% C.I. 1.05–2.43)	0.027
Former smokers				
Controls	11	50.9 ± 6.3	1	
Workers exposed to wood dust (alone)	17	67.2 ± 16.8	0.93 (95% C.I. 0.48–1.78)	0.829
Woodworkers exposed to VOCS	8	96.6 ± 21.1	1.52 (95% C.I. 0.71–3.26)	0.211
Current smokers				
Controls	26	59.8 ± 5.1	1	
Workers exposed to wood dust (alone)	34	60.8 ± 8.1	0.78 (95% C.I. 0.58–1.14)	0.211
Woodworkers exposed to VOCS	16	158.4 ± 29.3	2.33 (95% C.I. 1.45–3.70)	0.0003

^a^*p*-values (Test of Wald) were adjusted for age.
